# Sensor-based augmented visual feedback for coordination training in healthy adults: a scoping review

**DOI:** 10.3389/fspor.2023.1145247

**Published:** 2023-06-29

**Authors:** Heinz Hegi, Jakob Heitz, Ralf Kredel

**Affiliations:** Institute of Sport Science, University of Bern, Bern, Switzerland

**Keywords:** sensor-based, augmented feedback, visual feedback, motor learning, coordination training, autonomous training, design guideline

## Abstract

**Introduction:**

Recent advances in sensor technology demonstrate the potential to enhance training regimes with sensor-based augmented visual feedback training systems for complex movement tasks in sports. Sensorimotor learning requires feedback that guides the learning process towards an optimal solution for the task to be learned, while considering relevant aspects of the individual control system—a process that can be summarized as learning or improving coordination. Sensorimotor learning can be fostered significantly by coaches or therapists providing additional external feedback, which can be incorporated very effectively into the sensorimotor learning process when chosen carefully and administered well. Sensor technology can complement existing measures and therefore improve the feedback provided by the coach or therapist. Ultimately, this sensor technology constitutes a means for autonomous training by giving augmented feedback based on physiological, kinetic, or kinematic data, both in real-time and after training. This requires that the key aspects of feedback administration that prevent excessive guidance can also be successfully automated and incorporated into such electronic devices.

**Methods:**

After setting the stage from a computational perspective on motor control and learning, we provided a scoping review of the findings on sensor-based augmented visual feedback in complex sensorimotor tasks occurring in sports-related settings. To increase homogeneity and comparability of the results, we excluded studies focusing on modalities other than visual feedback and employed strict inclusion criteria regarding movement task complexity and health status of participants.

**Results:**

We reviewed 26 studies that investigated visual feedback in training regimes involving healthy adults aged 18-65. We extracted relevant data regarding the chosen feedback and intervention designs, measured outcomes, and summarized recommendations from the literature.

**Discussion:**

Based on these findings and the theoretical background on motor learning, we compiled a set of considerations and recommendations for the development and evaluation of future sensor-based augmented feedback systems in the interim. However, high heterogeneity and high risk of bias prevent a meaningful statistical synthesis for an evidence-based feedback design guidance. Stronger study design and reporting guidelines are necessary for future research in the context of complex skill acquisition.

## Introduction

In the last decades, technological progress has brought about a multitude of competitively priced sensor devices for recording and analyzing human movement in real time. In the context of sports and exercise, this development led to a variety of commercial products leveraging sensor-based augmented feedback applied in domains ranging from physical activity monitoring to classical strength and endurance training to exergaming and even motor-skill learning (1). Such autonomous technological solutions promise to be an efficient and (cost-)effective complement to classical instructor-led interventions and are therefore marketed aggressively for home training, but also for fitness centers and even for clinical use in physical therapy and rehabilitation. The prevalence of human trainers and their obvious benefits in all kinds of sport training alone form strong indicators that such sensor-based augmented feedback training (SAFT) systems may also provide advantages in the aforementioned domains while tackling already prevailing and in the future intensifying cost and personnel capacity issues. Therefore, further investigation of potential benefits but also harms of sensor-based augmented feedback seems necessary.

In general, SAFT systems are intended to foster sensorimotor learning, a process which brings about a relatively permanent improvement in the capability of a person to perform a sensorimotor skill (2). From a theoretical perspective on motor control and learning, four principal sensorimotor learning mechanisms can be distinguished, which extend Newell's well-known task-space landscape metaphor (3) and were first elaborated by Hossner, Kredel, and Franklin (4)—namely, task-space formation, differentiation, exploration and (de-)composition. It quickly becomes apparent that SAFT systems can foster sensorimotor learning during all these stages. First, SAFT systems can assist novices during task-space formation, where learners need to identify basic functional task structures. As Hossner and Zahno (5) state, this process can be enhanced by (i) providing task-goal related instructions, (ii) following appropriate schedules, or (iii) introducing part-whole training. Not only can SAFT systems provide this information in a reliable and systematic manner, moreover, they can analyze the learner's compliance based on the gathered sensor data and adapt to potential deviations. Second, during task-space differentiation, learners start paying attention to less salient task parameters, thus increasing the dimensionality of the task-space. SAFT systems can support this process by inducing controlled amounts of variance, e.g., by increasing difficulty or augmenting errors. This contributes to optimally structured learning contexts that promote the identification of additional task-relevant control variables while, at the same time, assuring the exploration of the continuously evolving task subspaces. Third, SAFT systems allow to point the learner towards better task solutions during task-space exploration and therewith promote a systematic escape from local optima. According to Hossner, Kredel, and Franklin (4), this can be achieved by avoiding repetitive, blocked practice of task variants, which fosters a stronger representation in memory [cf. the reconstruction hypothesis (6)] and facilitates an interpolation of the explored support points of the task space [cf. the elaboration hypothesis (7)]. Fourth, such a well-explored task space can be expected to allow for a better transfer of sub-spaces containing movement structures into the context of different tasks. Consequently, during task-space (de-)composition, learners need to be supported in identifying functional (sub-)structures in their task spaces that can be potentially applied outside the current motor task (5). Applying the above reasoning again, as SAFT systems allow for a systematic variation of specific, functionally relevant control variables while keeping others constant, their application can promote structure detection and therefore (sub-)space identification. Moreover, decomposing a task into such transferrable sub-structures may allow to train those in isolation, increasing the quality of the building blocks independent from training the whole task (4). Functionally relevant task-space decomposition would additionally allow to start task-space exploration with a well-educated guess, consequently changing the learning of completely novel tasks to a transfer of functionally fitting subspaces from previous experience (5). With its fine granularity on sensory motor learning mechanisms, this theoretical framework has the potential to guide the conceptual design of SAFT systems to ultimately benefit sensorimotor learning.

Despite all potential benefits, a major challenge remains for a successful application of SAFT systems to sensorimotor learning: Finding appropriate approaches to guide the learner to specific regions of the task space, in other words, defining the optimal type and amount of instruction and feedback for the current experience level of the individual learner. Well established approaches in sports practice can be differentiated by the amount of structure provided during the learning process. They form a continuum between unsupervised and supervised learning regimes.

On one end of the continuum, and like unsupervised learning, (unguided) discovery learning builds on the self-organized search behavior by the learners, assuming that they can find their optimal task solution better than any external observer [e.g., Vereijken and Whiting (8)]. When targeting specific mechanisms of motor learning as sketched above, this approach seems particularly suited to exploit inherent variability, while a systematic addressing of specific regions of the task space seems limited.

Applying a rather prescriptive approach, located at the other end of the continuum, those specific regions might be targeted more easily by explicitly instructing the learner, ideally in the form of desired sensory consequences. Those instructions are thought to generate sensorimotor imagery together with the desired action consequences and therefore provide sufficient input to the motor system to parametrize the movement (4). While older research found larger detrimental effects due to raised psychological demands for explicitly learned skills (9), in a recent review, Kal et al. (10) did not find clear disadvantages in their descriptive synthesis. They therefore explicitly encourage employing both approaches in practice based on their appropriateness for the task and learning challenge at hand. Nevertheless, applying instructions and feedback excessively may introduce artificial feedback-specific dimensions to the task space which provide highly precise information for movement parametrization. From a Bayesian perspective, the estimations throughout the learning process would be dominated by those artificial dimensions over noisier, task-relevant dimensions. However as soon as feedback is removed, the artificial dimensions do not provide meaningful information anymore, preventing the sensorimotor system from finding a good solution. This phenomenon is known as the guidance effect (11, 12). Even if this effect does not necessarily generalize to more complex tasks [e.g., (13–16)], considering the general mechanism seems sensible.

In their 2002 review, Wulf and Shea (14) concluded that principles derived from simple skill learning do not necessarily generalize and more intensive research on complex skills is required to advance motor learning theory and to adequately inform practice. Since then, most research has been investigating augmented feedback very broadly [cf. Sigrist et al. (17)]. Neglecting given instructions and experience levels while including multiple modalities, mixed populations, and simpler movement tasks in medical settings generally results in a very heterogenous set of outcomes not allowing for a clear-cut synthesis of the results. The combination of these factors may have contributed to the ambiguous result patterns in prior research on augmented feedback in motor skill learning.

In this review, an approach involving a restrictive search purview has been employed to increase the homogeneity of the included research. Diminished health, older age, or different levels of motor development may affect motor learning and the optimality of developed strategies, so we restricted target population to healthy, non-elderly adults. When it comes to the task complexity-dependent effect of feedback, it is still unclear whether it should be regarded as a binary question of simple movements vs. complex movements, or rather as a spectrum. We thus opted for a conservative definition of complex movements that involves postural control and multi-joint movements, further limiting the considered experiments to sports-related coordination training interventions with such complex movement tasks. A previous review on the potential impact of different feedback modalities and parameters has concluded that vision was the most investigated modality (17), which can be enforced from an implementational viewpoint due to the ubiquity of electronic screens in digital technologies and existing training devices. By focusing on visual feedback as the largest body of evidence only, we expect to maximize the review's synthesis potential. To sum up, the objective of our scoping literature review is thus to provide the basis for informed feedback design and to provide guidelines for the development of future autonomous visual SAFT systems for sports-related settings to maximize the training benefits derived from such feedback. More specifically, we approach this objective by addressing the following goals:

i. Aggregate results pertaining to similar feedback regimes to provide an overview of the findings in relation to these choices.
ii. Outline what visual feedback regimes have been considered in sports-related research.
iii. Compile the recommendations made in these studies regarding visual feedback regimes.

## Methods

We followed the PRISMA Extension for Scoping Reviews (PRISMA-ScR) (18) without prior registration of a formal review protocol. A research librarian advised the investigators in the selection of the databases and the formulation of the search strings. In accordance with the recommendations of the Interim Guidance from the Cochrane Rapid Reviews Methods Group (19), the three electronic databases Embase, PubMed, and Cochrane Central were searched to cover a comprehensive basis of the available literature. The last search on each database was carried out on the 17th of October 2022 by one investigator. The search strings consisted of a conjunction of disjunctions, grouped into the following four inclusion criteria (with *NEAR/10* meaning that the respective keywords need to be closer than ten words):

• **Feedback:** (“knowledge of performance” OR “knowledge of results” OR ((augment* OR external OR extrinsic OR kinetic* OR kinematic* OR motion) NEAR/10 (feedback OR biofeedback)))
• **Coordination:** (performance OR motor OR movement OR skill* OR coordination OR neuromuscular OR techni* OR athlet* OR sport*)
• **Training:** (training OR acquisition OR improvement OR learning OR athlet* OR sport*)
• **Visual:** (visual* OR display* OR screen OR perceptual*)

The search was limited to articles published in peer-reviewed journals and always covered abstracts. If the database interface permitted a combined search with titles and keywords, then these were also included. Where possible, filters were set to exclude reviews and study registrations and to only consider intervention studies. If this was not possible, the filtering process was performed manually in the screening phase. There was no restriction to sensor-based feedback in the search terms because such specifics of the methodology may be missing in the abstract.

The screening procedure consisted of two phases: The first phase was based on abstracts, titles, and keywords, while the second phase considered the full-text articles. In both phases, two screeners read all records. After the first phase, 52 items had conflicting verdicts, which were then discussed on a one-by-one basis until a consensus was reached between both screeners. After the second phase, all results were discussed to verify the final selection. Studies in languages other than English were excluded, as well as studies older than 30 years (publication year 1991 or earlier) as sensor-based real-time feedback was practically unavailable before. Studies were excluded if they did not include a complex sports-related coordination task with sensor-based visual feedback or did not have at least one group of healthy, non-elderly adult participants. The general rationale behind these criteria was mostly based on the theoretical aspects that were discussed in the introduction. A practical explanation with the resulting concrete differentiations in the screening procedure is given here:

• Sensor-based feedback:
  Our goal was to restrict the purview to feedback that was generated in an automatic and objective manner, as opposed to, e.g., human augmented feedback from coaches or peers. This decision has some unintuitive consequences: Video-feedback was included, because it is technically a sensor, while other visual feedback generated by electronic devices such as laser pointers was not included.
• Visual feedback:
  By focusing on one feedback modality, we hope to attain more consistent results. However, we still included studies that added other feedback modalities to the provided visual feedback if the visual feedback was clearly in the focus. Other intervention groups with different feedback modalities or no feedback at all were considered as control groups for the data extraction.
• Healthy, adult, non-elderly population:
  Disorders, diseases, and age could affect motor learning mechanisms, because these factors might alter the optimality of specific movement solutions and because cognitive maturity or decline might affect motor learning. Thus, as a rather conservative boundary, we only considered participants that are between 18 and 65 years. If a study involved at least one group of participants that fully satisfies these criteria, then the study was included even if other groups were considered in the study. In that case, all groups not satisfying these criteria were ignored during the data extraction.
• Sports-related, complex sensorimotor tasks:
  We expected participants to have a different mindset in sports-related training compared to medical settings. Compared to sports, interventions targeting activities of daily living (ADL) generally have a different focus, and, in turn, a potentially different feedback objective. Therefore, we excluded ADL and simple balancing tasks.
  We purposefully drew the line between simple and complex tasks rather conservatively so that any study lying between clearly complex and clearly simple tasks was excluded as well. This should ensure that possible negative outcomes stemming from tasks that were not quite complex enough are fully avoided in the synthesis of outcomes, but it is in no way meant as a definition for what constitutes a complex movement task. Tasks which required active control of only one single joint were excluded, as well as bimanual tasks such as reaching, pointing, or sequencing. On the other hand, rowing studies were included despite the seated position if the correct execution of the task required coordination of leg, hip, and trunk movements in addition to the movement of the arms.

After the full-text screening, included studies were categorized into three distinct groups according to the applicability of their results for a potential synthesis. First, if a study reported on the difference between pre- and post-tests for intervention and comparable control groups, with all participants satisfying our population inclusion criteria, then it was categorized as reporting a *training effect*. This category has the potential to indicate how visual feedback design affect retention effects.

For the control group to be considered as comparable, we required that it was different from the intervention group, both regarding participants (i.e., a distinct set of people) and the provided feedback: the control must have either no feedback, a different feedback modality, or also visual feedback but with a relevant change to the way it is designed or administered. Furthermore, the feedback must be withdrawn during testing for all groups to ensure that the measured effects stem from changes in the motor skill in the original task. The measured effect must therefore constitute actual learning and not just a temporary effect caused by the task difference brought about by the given feedback. Second, a study that compares feedback trials with no-feedback trials was categorized as reporting *immediate effect* of feedback. The control can again consist of no-feedback, a different modality, or visual feedback with some aspects changed. Contrary to the first category, these studies must necessarily include tests or measurements with feedback. The control group can either be a different group of participants like in the first category, or alternatively the same group under different feedback conditions in a within-subject design. Therefore, whereas the first category required at least two groups of participants satisfying our population inclusion criteria, one such group was enough to categorize the study as reporting on immediate effects. Third, all other studies were only deemed relevant from a *design-only* perspective, with the focus on the design choices rather than their results. To be included in this category, studies still had to satisfy our inclusion criteria, but they either had exactly one participant group satisfying our population criteria and no within-subject design, or they had multiple participant groups that were not comparable because they did not differ in the administration of the visual feedback (for example only differing in other feedback modalities administered in conjunction with visual feedback).

For the structured data extraction, two investigators extracted information and co-edited the results into a table. Conflicting table entries were discussed until a consensus was reached. The table was then stratified so that all entries follow common nomenclature, and further condensed into the two final, more concise tables presented in this article. The study characteristics were summarized in a first table (Table 1), where the columns broadly describe the category, the task and its goal, the intervention, and the participants for each study. A second table (Table 2) was split into the three study categories (training effect, immediate effect, design-only) by horizontal lines, using multiple rows for reports including multiple studies, depicting details of the outcomes and the visual feedback regimes for each study. For each main outcome of the studies in the training effect category, at most one post-test (PT) directly following the last intervention session, one short-term retention test (RT1) at least 1 day after the last intervention session, and one long-term retention test (RT2) were considered, each of which is represented in a different column. Potential additional retention tests were discarded because they would only describe the pattern of depreciation over time in more detail. Since the time effect of the interventions in these studies cannot be clearly separated from the immediate effect of the feedback, measurements during the intervention phase were not considered for this study category. Conversely, such immediate tests (IT) were considered for the studies in the immediate effect category, where the focus is not on the effect of the intervention over time but rather on how the feedback affects performance at the instant when it is applied. Finally, no outcome measures were reported for the design-only category because these studies are only relevant for the overview of feedback regimes in the literature, i.e., goal (ii) of this review. The outcomes were represented by arrows indicating whether participants in the visual feedback intervention performed significantly better (⇑), significantly worse (⇓), or not significantly different (⇔) when compared to the control group. For the training effect category, these reported effects always refer to the learning rates or the change from baseline to post- or retention tests (PT, RT1, RT2), in other words group-by-time interaction effects. Conversely, immediate effect studies always refer to the group effects measured (IT), while potential time effects were discarded. Other tests in the respective categories were not reported in the table. In case of differing outcomes, effects for multiple main outcomes were represented separately by splitting them into multiple lines while comparisons to multiple control groups were separated by commas. Multiple visual intervention groups were addressed by prefixing these comparisons with a letter assigned to the different groups (for more details on the chosen nomenclature, refer to the note below Table 2). The chosen intervention groups could have multimodal feedback, but visual-only groups were preferred if available, in which case additional multimodal groups would be disregarded in the reporting of outcomes.

**Table 1:** Overview of tasks, goals, interventions, and population characteristics.

Identifier	Type	Task	Goal	Duration	Sessions	Groups	*N*	Age	Sex	Experience
Benjaminse et al. (20)	TE	Sidestep	Reduce peak knee forces	1	1	3	90	24.6 ± 4.4*	X	Advanced
Chan et al. (21)	TE	Treadmill Running	Soften footfalls	14	8	2	320	18–50	X	Intermediate*
Ericksen et al. (22)	TE	Jumping	Stick the landing	1	1	3	36	20.7 ± 2.3*	F	Beginner
Gilgen-Ammann et al. (23)	TE	Running	Reduce ground contact time	28	8	3	30	31.0 ± 7.5	X	Advanced
Mononen et al. (24)	TE	Shooting	Maximize accuracy	28	12	4	34	20.4 ± 1.8	M	Intermediate
Mulloy et al. (25)	TE	Fencing Lunge	Maximize propulsion, keep sequencing	180	6	2	32	18–40	X	Novice
Nagata et al. (26)	TE	Jump Squats	Increase lifting velocity	28	7	4	37	19–22	M	Advanced
Nekar et al. (27)	TE	Squats	Maintain proper form	28	12	4	48	18–35	M	Beginner
Post et al. (28)	TE	Golf Chipping	Hit target, maintain form	1	1	2	44	21.8 ± 1.3	X	Novice
Rauter et al. (29)	TE	Rowing	Follow reference	2	2	5	40	19–32	X	Novice
Rauter et al. (30)	TE	Rowing	Match target movement	2	2	2	16	27.7 ± 1.9	X	Novice
Rucci and Tomporowski (31)	TE	Hang Power Clean	Maximize power output	28	7	3	17	18–22	F	Intermediate
Sigrist et al. (32)	TE	Rowing	Match target movement	3	3	4	35	28 ± 3.7	X	Novice
Todorov et al. S1 (33)	TE	Table Tennis Return	Hit target through barrier	1	1	3	42	NA	X	Novice
Todorov et al. S2 (33)	TE	Table Tennis Return	Hit target through barrier	3	3	2	18	NA	X	Novice
Viitasalo et al. (34)	TE	Shooting	Maximize accuracy	84	36	4	30	37.5 ± 11.3*	M	Beginner
Anson et al. (35)	IE	Treadmill Walking	Reduce trunk variability	1	1	1*	10*	22.6 ± 4.9	X	Intermediate
Eriksson et al. (36)	IE	Treadmill Running	Adjust running technique	1	1	1	20	28.4 ± 6.4	X	Advanced
Hamacher et al. (37)	IE	Walking	Achieve a balanced gait in frontal plane	1	1	1*	15*	45–65	F	Intermediate
Jones et al. (38)	IE	Ergometer Cycling	Increase performance	21	4	2	20	35.5 ± 6.5*	M	Advanced
Koritnik et al. (39)	IE	Stepping	Match reference	1	1	2	23	23–30	X	Intermediate
Washabaugh et al. (40)	IE	Treadmill Walking	Use full range of motion of knee joint	1	1	1	13	21.0 ± 2.5	X	Intermediate
Weakley et al. (41)	IE	Back Squat	Maximize concentric power	14	4	1	12	21.8 ± 0.9	M	Intermediate
Sigrist et al. (42)	DO	Rowing	Match target movement	2	2	3	24	26.1 ± 3.0	X	Novice
Teng et al. (43)	DO	Treadmill Running	Increase trunk flexion	28	4	1	12	23.3 ± 3.8	X	Intermediate
Teran-Yengle et al. (44)	DO	Treadmill Walking	Avoid knee hyper-extension	1	1	1	17	26.6 ± 5	F	Intermediate

The studies are specified by category (type: TE, training effect; IE, immediate effect; DO, design-only), task, goal, characteristics of the intervention (duration in days, sessions, groups), and population: *N *= number of participants, age (years, either as range or as *M *± SD), sex (M, male; F, female; X, mixed), and experience. NA means not available.

* Adjusted by review authors (only counting healthy, adult, and not elderly participant groups; aggregated age; different definitions for experience levels).

**Table 2:** Overview of dependent variables, applicable effects, and feedback regimes.

Identifier	CG	Outcome Measures	IT	PT	RT1	RT2	Feedback Measures	Content	KP	KR	C	T	R	F [%]
Benjaminse et al. (20)	Coach, No	Segment Angle (Trunk) Biomechanical Measures	NA	–	⇑, ⇔ ⇔	–	Scene^T^	Video	X	–	–	X	X	100
Chan et al. (21)	No	Ground Reaction Force Injury Occurrence	NA	⇑ –	–	– ⇑	Force^T^	Plot	X	–	X	–	–	≈67^fH^
Ericksen et al. (22)	Coach^M^, No	Joint Angles (Hip, Knee) Ground Reaction Force	NA	⇔, ⇑	–	–	Segment Position^T^	Segments + Line^M^	X	–	X	X	X	100
Gilgen-Ammann et al. (23)	No	Ground Contact Time	NA	–	⇑	–	Mean Time	Bar + Num.	X	–	–	X	X	100H
Mononen et al. (24)	Visual	Score + Score Variability Directional Errors	NA	–	F: ⇑; P: ⇔ ⇔	⇔	Aiming-Point^T^ & Position + Score	Target + Trace & Target + Num.	X –	– X	–	X	– X	F: 100; P: ≈50 100
Mulloy et al. (25)	No	Angular Velocities (Hip, Knee, Ankle)	NA	–	⇔	–	Maximum Angular Velocity + Timing	Color Bar Chart	X	–	–	X	X	≈70
Nagata et al. (26)	No, Coach	Barbell Velocity	NA	⇔	–	⇔	Scene^T^ Mean Velocity	KP: Video; KR: Num.	X –	– X	–	X	–	100
Nekar et al. (27)	Coach^M^, Visual, No	Knee Extension + Balance Knee Flexion Flexibility	NA	⇔, ⇑, ⇑ ⇔, ⇔, ⇑ ⇔	–	–	Scene^T^	AR^M^	X	–	–	X	X	100
Post et al. (28)	Visual^Y^	Accuracy Form Score	NA	–	⇔	Transfer: ⇑	Scene^T^	Slow-Motion Video + Video	X	–	–	X	X	100^S^
Rauter et al. (29)*	Haptic	Spatial Error Velocity Error	NA	–	⇔	–	Position^T^	Oar Trace	X X	– –	X –	– X	X X	≈70
Rauter et al. (30)	Visual^Y^	Spatial Errors	NA	–	⇔	–	Position^T^	Trace^M^	X	–	X	–	X	≈70E
Rucci and Tomporowski (31)	Verbal	Strength + Power Form Score	NA	⇔ ⇓	–	–	Scene^T^	Video	X	–	–	X	–	100
Sigrist et al. (32)	Haptic, Audio	Absolute Angular Error Scaling Error Rotation Error Velocity Error Movement Variability	NA	–	⇔ ⇔ C: ⇔; T: ⇑ C: ⇔; T: ⇔, ⇑ C: ⇔, ⇑; T: ⇔	C: ⇔; T: ⇔, ⇑ ⇔ ⇔, ⇑ ⇔ ⇔	Position^T^ + Orientation^T^	C: Oar; T: Oar + Trace	X	–	X –	– X	X	C: 72 T: ≈72^S^
Todorov et al. S1 (33)	Coach^M^	Accuracy Score	NA	⇑	–	–	Position^T^ & Score	Trace & Num.	X X	– –	X –	– X	X X	100
Todorov et al. S2 (33)	Coach	Accuracy Score Trajectory-Distance Score	NA	⇑	–	–	Position^T^	Trace & Num.	X	–	X	X	X	≈33
Viitasalo et al. (34)	Visual, Coach^M^	Accuracy	NA	⇔	–	–	Aiming-Point^T^ & Forces & Scene^T^ & Position + Score	Target + Trace & Num. & Video^M^ & Target + Num.	X X –	– – X	– – –	X X X	– X –	≈14 ≈17 100
Anson et al. (35)	No	Low-Frequency- Translational Variance Various Gait Parameters	⇑ ⇔	NA	NA	NA	Position^T^	Target + Dot	X	–	X	–	–	100
Eriksson et al. (36)	Audio^W^	Vertical Displacement Step Frequency	⇔	NA	NA	NA	Positions^T^ + Mechanical Power^T^	Bar Chart	X	–	X	–	X	100
Hamacher et al. (37)	No^W^	RoM, Inclination (Pelvis) RoM, Inclination (Trunk)	⇔ ⇑	NA	NA	NA	Segment Angles^T^	Avatar + Axes	X	–	X	–	X	100
Jones et al. (38)	Visual^W^, Visual	Time, Speed, Power, Perceptual and Physiological Measures	⇑, ⇔	NA	NA	NA	Position^T^ Distance^T^	Avatars Num.	X	X	X	–	X	100
Koritnik et al. (39)	Visual	Spatial & Temporal Adaptation	⇑	NA	NA	NA	Joint Angles^T^	Avatar	X	–	X	–	X	100
Washabaugh et al. (40)	No^W^	Joint Angle + Aftereffects (Knee), Muscle Activation	⇑	NA	NA	NA	Joint Angles^T^	Bar Chart	X	–	X	–	X	100
Weakley et al. (41)	Coach^W^, Coach^W^, No^W^	Barbell Velocity	⇔, ⇔, ⇑	NA	NA	NA	Mean Velocity	Num.	X	–	–	X	–	100
Sigrist et al. (42)*	NA^W^	Spatial & Temporal Errors (Angle + Angular Velocity)	NA	NA	NA	NA	Position^T^	Oar^M^ Trace^M^	X X	– –	X –	– X	X X	≈70^E^
Teng et al. (43)	NA^W^	Kinematics (Trunk), Joint Kinetics (Hip, Ankle), Automaticity	NA	NA	NA	NA	Segment Angle^T^ Score	Dots Num.	X X	– –	X –	– X	X –	≈50^f^^H^
Teran-Yengle et al. (44)	NA^W^	Joint Angle (Knee)	NA	NA	NA	NA	Joint Angle^T^	Plot	X	–	X	–	X	≈50

The studies are ordered by category (first training effect, then immediate effect, then design-only, see Table 1). The ‘CG' column specifies the type of control groups (instruction/feedback conditions: No, Visual, Haptic, Audio, Coach, ^M^ = multimodal, ^Y^ = yoked control group, ^W^ = within-subject comparison). Interaction effects of feedback for immediate testing (IT), post-test (PT), first retention test (RT1), and last retention test (RT2) are shown by arrows. ⇔: no significant difference between intervention group (IG) and control group (CG), ⇑: IG significantly better, ⇓: IG significantly worse; if effects are identical for multiple outcome measures and/or control group comparisons, they are summarized by one arrow, in case of differing effects: A comma ',' splits control groups, a semicolon ';' splits feedback groups, letters are assigned if multiple visual feedback regimes were applied (F, full; P, partial; C, concurrent; T, terminal). NA means not applicable or not reported. ^T^ indicates that the feedback measure had a time-component. In addition to feedback measure and graphical content of the visual feedback display (properties are linked by a plus sign ‘+' if presented simultaneously, while an ampersand ‘&’ links multiple quantities separately shown), the following properties of the feedback regime are shown: knowledge of performance (KP), knowledge of results (KR), concurrent (C) or terminal (T) feedback, whether a reference (R) was available or not (X = applied, - = not used), and feedback frequency (F in %). Frequency is either given as fixed percentage of trials/time, as overall average percentage for fading (^f^), or as maximum allowed percentage for self-selected (^s^) and error-based (^E^) regimes, without considering reported additional home-exercise (^H^).

* Rauter et al. (29) utilized the same visual feedback group's data as the unimodal visual group in Sigrist et al. (42), so their feedback regime was clearly identical without adding to the available evidence. We instead listed the multimodal audiovisual feedback group for Sigrist et al. (42).

Study populations were classified according to our estimation of their experience in performing the specific movement task. This classification does not necessarily coincide with the one used in the corresponding reports, which were usually based on levels of competition of the recruited participants instead. We classified participants as *Novice* if they had likely no prior experience with the task. Further, *Beginner*, *Intermediate*, and *Advanced* refer to some experience, regular experience, and expert-level experience with the task, respectively.

The qualitative extraction of the recommendations made in the literature was a less structured process. The discussion and conclusion sections of the included studies were screened for statements that we deemed relevant and generalizable for informing future feedback design. Such statements were only extracted if they satisfied two additional conditions: they were based on the results found in the study (as opposed to other referenced research), and they went beyond descriptions and explanations of the outcomes. Two reviewers marked potential candidate passages in the text, and one reviewer then made the decision whether they should be picked up in the result section of this review. The intention was to include only the most important statements in a concise overview.

Finally, one investigator performed a risk of bias assessment using the risk-of-bias tool for randomized trials (ROB 2) (45) for each study in the training effect category. The rationale for this assessment was to evaluate the strength of evidence that a potential meta-analysis could provide in a systematic review of this research topic.

## Results

### Study selection and data extraction

**Figure 1:**
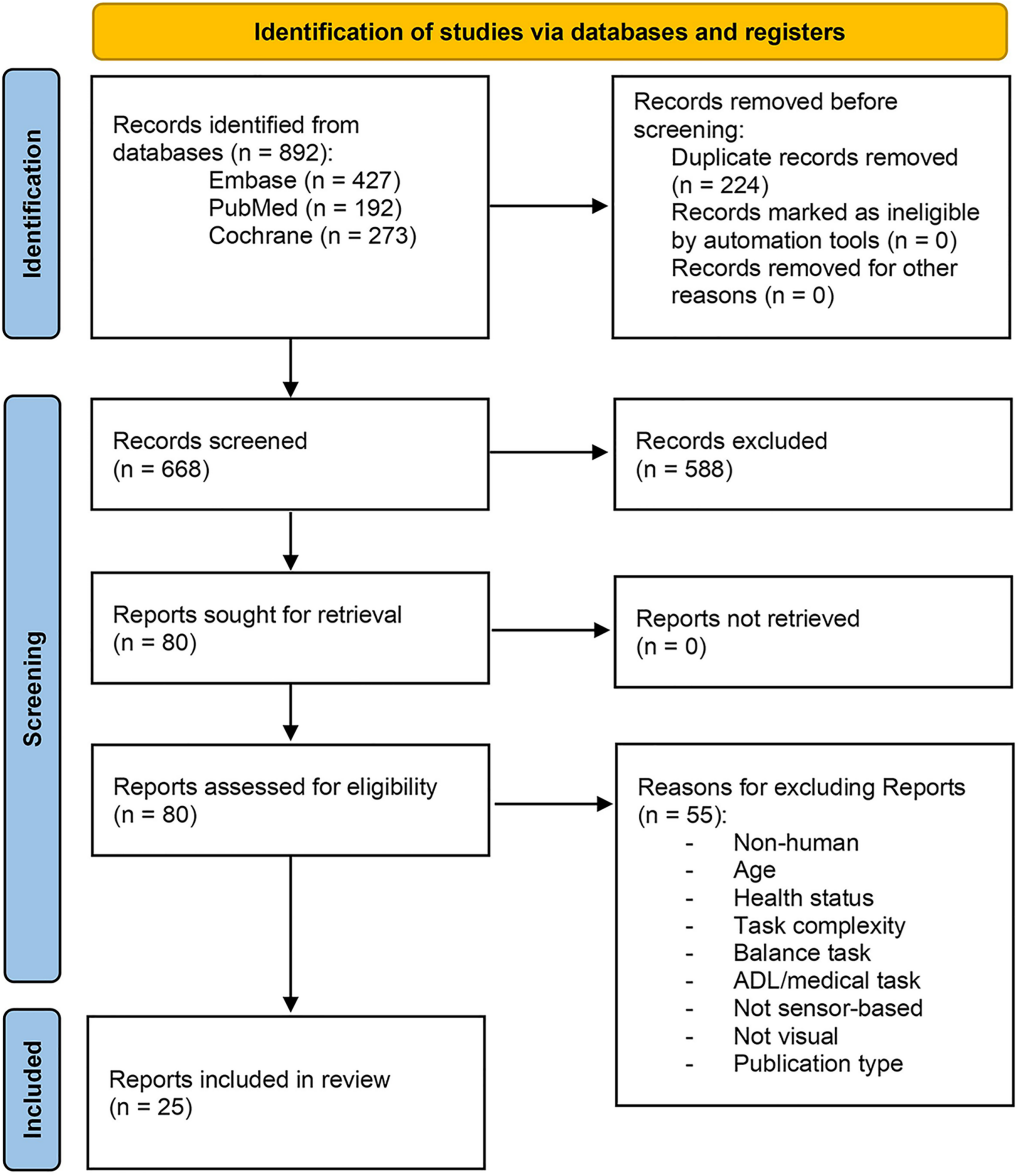
PRISMA 2020 (46) flow diagram: overview of the study selection process consisting of database searches, abstract screening, and full-text assessment.

The initial literature search identified 892 records from three databases (Figure 1) (46). After removing 224 duplicates, 668 distinct records remained. From these, we excluded all records that did not satisfy the criteria specified in the methods section: 588 records were excluded in the abstract screening stage and 55 reports during the full-text screening, leading to 25 reports included in the final dataset. 15 of these 25 reports measured training effects of visual feedback, but one report consisted of two empirical studies, so in total 16 studies were assigned to the training effect studies. The remaining 10 reports were not eligible for the training effect category because some only had one intervention group satisfying our population criteria (7 reports), no post-intervention tests without feedback were performed (2 reports), or because the control groups differed in other feedback modalities without affecting attributes of the visual feedback (1 report). Of these 10 reports, seven measured performance under different visual feedback conditions and were thus eligible for the immediate effect category, featuring five within-subject designs, one between-subject design, and one with both within- and between-subject comparisons. The remaining three reports did not compare immediate performance under different visual feedback conditions, but instead reported training effects over time for a single group (2 reports) or had control groups that all received the same visual feedback (1 report). All 26 studies of the 25 reports and their characteristics deemed relevant for this review are summarized in Table 1 (population and intervention) and Table 2 (dependent variables and feedback).

Several small adjustments were made during the data extraction process. Two studies incorporated groups of participants that did not match our population criteria (35, 37), these groups were subsequently ignored in the data extraction. Multimodal groups receiving visual feedback were disregarded in three studies (31, 39, 42) in Table 2 because visual-only intervention groups and non-visual control groups were available. Rauter et al. (29) designated the visual feedback group as control group, but for our purposes this constitutes the intervention group, with the haptic feedback groups serving as control instead. In three studies (23, 24, 34), one “true” control group, in which the participants received no intervention at all, was disregarded in Table 2. An item of concern was that Rauter et al. (29) and Sigrist et al. (42) seemed to share the same visual-only feedback group, i.e., only one unique dataset was gathered for both studies. The visual-only feedback group is therefore counted twice in the columns of Table 1 that concern study participants. This group was assigned to Rauter et al. (29) as the main intervention group in Table 2 so that it could be counted for group comparisons in the training effect outcomes. Because Sigrist et al. (42) is in the design-only category, the same group is not relevant for group comparisons here, so this group was ignored for this study in Table 2 to avoid over-representation of the same feedback regime. Instead, the otherwise similar multimodal group was considered as the main intervention group in Sigrist et al. (42).

### Populations and intervention formats

Most studies (18 out of 26) had a relatively small group size with less than 15 participants per intervention arm (Table 1 columns “Groups” and “N”). The exceptions were Benjaminse et al. (20) with 30, Chan et al. (21) with 160, Mulloy et al. (25) with 16, Post et al. (28) with 22, Eriksson et al. (36) with 20, Hamacher et al. (37) with 15, and Teran-Yengle et al. (44) with 17.

Instructions were often implicit to the task, e.g., trying to hit a target implicitly conveys the desire to increase accuracy, which was the goal in 5 out of 26 studies. Increasing physiological power output was the objective in 5 studies. More nuanced instructions consisted of following a target movement (5 studies), reducing joint strain (2 studies), or a direct adjustment to the movement technique (11 studies). Two studies explicitly combined the performance goal with the demand to maintain proper technique.

When classifying the studies according to their intervention schedule, 10 studies lasted for less than 1 day, encompassing a single session, while 5 studies lasted between 2 and 3 days with 2–3 sessions. Nine studies lasted between 2 and 4 weeks with 4–12 sessions; the remaining 2 studies lasted 12 weeks with 36 sessions and 6 months with 6 sessions, respectively.

### Utilized visual feedback regimes

The quantities used for the feedback mostly consisted of positions, joint angles, or forces relevant to the movement task, often coinciding with one of the dependent variables (cf. “Feedback Measures” and “Outcome Measures” in Table 2). These quantities were mostly measured using motion capture systems, cameras, force plates, and inertial measurement units. Todorov et al. (33) used an electromagnetic sensor to track paddle position and orientation. Nekar et al. (27) employed a mobile AR device. The rowing studies (29, 30, 32, 42) all utilized the same rowing simulator, which incorporated rope robots, motion capture, and wire potentiometers. The shooting studies (24, 34) employed an optoelectronic shooting system to detect the shot and to determine the relevant performance metrics. The shooting studies also included a trace of the point where the shooter was aiming at. Nagata et al. (26) used an optical encoder system to measure lifting velocity. In Eriksson et al. (36) and Weakley et al. (41), a position transducer measured the displacements and velocities, respectively. In Jones et al. (38), participants trained on a cycle ergometer. Participants in Washabaugh et al. (40) wore an exoskeleton that measured joint angles (while also applying the resistance for the movement task). Teng et al. (43) included the percentage of time spent in the desired parameter range as terminal feedback in addition to the concurrent joint angles measured by a motion capture system.

Knowledge of Performance (KP) feedback was given in every study, with four studies additionally including Knowledge of Results (KR) in the augmented feedback, but the timing of KP and KR feedback varied between studies. For KP, concurrent and terminal feedback was approximately equally common (in 16 and 17 studies respectively, shown in columns “C” and “T” of Table 2). One study, Sigrist et al. (32), reported a deliberate delay of terminal KP feedback during the trials: After feedback was requested by the participant, there was a 10 s delay, after which feedback was shown for the last 18 s of the movement. KR was given as terminal feedback in 3 of 4 studies, with only Jones et al. (38) giving concurrent KR feedback during their trials by displaying the total distance covered.

In 21 studies, some form of reference was incorporated to the visual feedback (as indicated in column “R” of Table 2). Possible forms of reference were ideal values or ranges (e.g., given as a line), a virtual avatar or a reference-oar performing the correct movement, or a split-screen video with another performance. Hamacher et al. (37) provided a reference by showing the current joint angles with the desired ranges overlaid on a virtual avatar of the participant. The data for the provided references was either sourced *a priori* (e.g., from recommendations or from experts showing the correct movements) or generated during the study from a participants' previous performances.

According to the following classification into four groups (plots, numerical, video, complex graphics), the 26 studies featured a total of 38 occurrences of graphical feedback visualizations (see column “Content” in Table 2). These visualizations varied in terms of graphical complexity and abstraction level, but no study tried to graphically convey more than three quantities at once and no study reported issues with the understandability of the graphics. In 12 studies the feedback was visualized by plotting it on a 2-dimensional plane. This was achieved with linked motion-capture marker-models (1 study), showing the trace of the movement on a plane (5 studies) or in a 3D virtual environment (2 studies), quantity-time plots (2 studies), dots on quantity-quantity plots (1 study), and markings on virtual bulls-eye targets (3 studies, two of which included aiming-traces). In 11 studies, numbers were represented as numerical values or vertical bars. A video recording of the participant was used in 6 studies, one of which involved augmented reality with graphical movement guidance. More complex graphical representations (9 studies) involved virtual avatars, a virtual copy of the training environment to show the trace in, or a virtual rowing simulator that included a virtual representation of the oar and other modalities (e.g., traces). In Jones et al. (38), the avatar was set on a virtual cycling track that graphically simulated a movement through space dependent on their cycling performance. Five studies (22, 27, 30, 34, 42) applied additional non-visual feedback in the visual feedback group, so the participants received multimodal feedback. Audio resulting from the simulation of water in the rowing studies (29, 30, 32, 42) were considered part of the immersion and not specifically marked as multimodal feedback in the table. Analogously, the virtual extension of the oar was not treated as visual feedback. All groups in all rowing studies received this audio and visual feedback.

A form of summary feedback (i.e., feedback that is not specific to a single movement execution) was used in Nagata et al. (26) by averaging over the whole set, and in Gilgen-Ammann et al. (23) by providing only the mean ground contact time over each interval run. Jones et al. (38) was the only instance where participants were deliberately deceived about the nature of the provided feedback: One group was told in one trial that the pacer (the reference avatar) showed their own performance from a baseline trial, without telling them that its speed was increased by 2%.

The reported frequency of each feedback schedule refers to the percentage of trials or time during the intervention phase in which participants had the opportunity to receive feedback (Table 2 column “F”). Test trials without feedback were treated the same as training trials without feedback if they consisted of the same movements. For the instantaneous effect studies, the frequency was generally 100% because there was no meaningful intervention phase to average over. The only possible exception is Jones et al. (38), which received a + 2% and a + 0% pacer as feedback for 25% of the time each, with the remaining 50% of the total time being reserved for baseline tests without pacer. In 18 studies, the feedback schedule was completely predetermined for at least one visual feedback group. In 8 studies, at least one group received visual feedback with other scheduling strategies. Fading feedback (a gradually decreasing frequency over the intervention duration) was used in Chan et al. (21) and Teng et al. (43). Self-selected feedback (providing feedback only upon request by the participant) was used in Sigrist et al. (32) and Post et al. (28). Self-selection led to variable feedback frequencies considerably different from the maximum possible frequencies, e.g., resulting in a mean frequency of 9% (range 2%–37%) compared to 100% possible in Post et al. (28). Error-based feedback (no or reduced visual feedback when performing below a certain error threshold) was used in three of the four rowing studies (29, 30, 42). Specifically, the trace was only drawn above the error threshold in Rauter et al. (29) and Sigrist et al. (42), and the transparency of the reference oar was increased with decreasing error, making it harder or even impossible to see. In Rauter et al. (30), visual feedback was provided if the spatial error was the dominant error, otherwise an auditive feedback was given for the velocity error instead. Three studies (21, 23, 43) explicitly reported that participants continued training outside the intervention sessions during the intervention period, at home or elsewhere. For these studies, the reported frequencies only refer to the training during the trials, other training (at home without feedback) was not taken into account.

### Effect of visual feedback on intervention outcomes

Using a vote counting approach, it is evident that the reported effectiveness of feedback varies a lot between studies (see Table 2, where votes are indicated by arrows). When interpreting these outcomes, it is crucial to also consider what exactly the intervention groups were compared against: Even the control groups showed high heterogeneity, which makes a fair comparison impossible. Only one study, Rucci and Tomporowski (31), reported that the visual feedback group showed worse outcomes than their control group, which received verbal feedback. Positive and no benefits are approximately equally common in the feedback and no-feedback conditions of the training effect studies. Even when looking only at the studies with the biggest group-sizes, the outcomes are mixed: Chan et al. (21) (160/group with fading) shows a clear benefit, Benjaminse et al. (20) (30/group with 100% feedback) and Mulloy et al. (25) (16/group with 70% feedback) show no benefit compared to no-feedback control groups, and Post et al. (28) (22/group) only shows a clear benefit in a transfer test. This pattern does not continue in the immediate effect studies, where feedback groups always outperformed no-feedback groups in at least one outcome measure. Otherwise, no clear pattern is visible regarding the time at which the tests were administered (“IT”, “PT”, “RT1”, and “RT2” in Table 2) or regarding specific feedback regime parameters. While the studies in the immediate effect category yielded proportionally more positive results than the training effect studies, this was not statistically tested either and no risk of bias assessment was performed for this category, so this may be due to publication bias. The tendencies shown in the tests of the training effect category are further relativized by the concerns shown in the risk of bias assessment.

Because of the high risk of bias and because the included studies are too heterogenous in their design and especially their outcome measures, a statistical synthesis of the findings was not conducted. The risk of bias assessment revealed high concerns for all experiments in the training effect category except for Ericksen et al. (22) (some concerns) and Nekar et al. (27) (low concerns). Chan et al. (21) was considered to have high concerns with regard to feedback effectivity since the control group did not receive instructions to “run softer” in the intervention (effectively resulting in no intervention instead of a no-feedback intervention). All other high concern evaluations are already determined by domain 1 (underspecified randomization process) and domain 5 (no information due to lack of prespecified analysis plan). Any synthesis based on these results would therefore suffer from a very low strength of evidence. Attributing outcomes (positive or non-significant) to movement tasks, experience levels, or specific feedback parameter choices is not warranted, since any purported effect could be attributed to random chance or bias (induced by the specific selection or grouping criteria) rather than a generalizable property of motor learning.

### Feedback regime recommendations from the literature

While Table 2 may serve as a basis to find similar research to consider in future SAFT studies, the remainder of this section is devoted to summarizing recommendations made by the authors of included studies. These recommendations are not necessarily based on hard evidence, i.e., significant study results with a low risk of bias, and instead represent a collection of informed opinions to pay attention to in the future scientific investigation of SAFT.

Benjaminse et al. (20) concluded that the ideal feedback modality might depend on gender, with males in their study benefiting from visual feedback, whereas females instead might benefit from different feedback modes. Anson et al. (35) further mentioned that visual processing is slower and therefore more amenable to slow movements when compared to other modalities. Additionally, larger movements may be easier to detect with visual feedback than smaller movement details. Sigrist et al. (32) suggested that the effectiveness of concurrent feedback may not only depend on the complexity of the movement task, but also the complexity of understanding the task requirements. They stressed that different feedback modalities have different strengths, and further explain that concurrent visual feedback may be more suitable for instructing complex movement, whereas haptic feedback should be used instead for temporal guidance. Sigrist et al. (42) also discussed modality-dependent benefits (sonification for temporal aspects, visual feedback for spatial aspects). However, no significant benefit of multimodal over unimodal feedback was found in the study. They concluded that the selective advantages may be determined by the exact design of the feedback rather than being inherent to the modality itself.

Benjaminse et al. (20) also mentioned that providing subject views from multiple angles might improve the outcome, but that feedback with high complexity can be detrimental. Post et al. (28), however, explicated that the instruction to focus on the (previously defined) critical features of the movement task may be sufficient to avoid overwhelming the learner with the information presented in video (even without offering a video-specific interpretation). Rucci and Tomporowski (31) corroborated other results according to which video feedback without additional cues has little effect on skill acquisition. They emphasized that regardless of the feedback modalities used to deliver feedback, it should provide information on how movement errors can be detected (instead of only directing the learners' attention to the error). This complements Mononen et al. (24), who argued that it might be difficult to establish a link between the received feedback and the corrections that should be made. Teran-Yengle et al. (44) mentioned that real-time feedback can provide the learner with specific information that is not available with intrinsic feedback, thus encouraging exploration and discovery of alternative movement solutions.

Jones et al. (38) concluded that the practical effects of challenging correct feedback as opposed to threatening deceptive conditions should be further explored, and that their effects may ultimately depend on the performance of the learner as well. Washabaugh et al. (40) emphasized the importance of using external motivational tools, such as feedback, to increase both learning and training intensity when intrinsic motivation is lacking. Weakley et al. (41) stressed the importance of providing encouragement and feedback during resistance training, and further noted that the extent of the benefit and the most successful way of providing such encouragement may also depend on individual characteristics, particularly the degree of conscientiousness. In this line of argumentation, Rauter et al. (29) suggested that future studies should tailor feedback to the experience of the participants, that feedback should be changed over the intervention time to prevent studies from becoming monotonous, and, moreover, that such changes have the potential to reduce the induced feedback-dependency (Note that these recommendations specifically concern the planning of feedback in studies and may not be meant as a direct recommendation for feedback in practice). Also, Sigrist et al. (32) recommended to combine multiple modes of feedback and to use an intelligent feedback strategy that individually tailors feedback to preferences, learning rates, error patterns, feedback susceptibility, and performance.

Ericksen et al. (22) explicitly cautioned against using the proposed feedback without first examining retention and transfer effects. Post et al. (28) mentioned that their study could represent an example where transfer may be a more sensitive test of learning, and that self-selected scheduling of split-screen feedback facilitates motor learning under the right circumstances. Todorov et al. (33) explained that the goal of their study was to show that augmented feedback can give an advantage in a difficult multi-joint movement, so the characteristics of augmented feedback in their study were chosen with that goal in mind. They stressed that this consequently does not constitute proof that all the choices made were required to achieve a significant performance benefit. In other words, the chosen conditions were deemed sufficient, but possibly not necessary.

The other reports only mentioned intervention effects and general explanations, but did not state explicit, generalizable feedback regime or study recommendations based on their results.

## Discussion

### Summary and limitations

We aggregated information about the intervention and visual feedback regimes utilized in 26 studies on training complex, sports-related sensorimotor tasks. We additionally presented the authors' recommendations concerning feedback regimes. In general, studies were practice-oriented and therefore compared considerably different interventions with various feedback regimes, without making generalizability of results for specific feedback parameters a priority. Despite our efforts to increase homogeneity by applying restrictive inclusion criteria, this remaining heterogeneity and the differences between the measured outcomes make it difficult to relate effects of single parameters changes over multiple studies. For the studies with multiple main outcomes, taking one as the main outcome for such a comparison would be an arbitrary choice with a high risk of introducing bias. Consequently, a statistical synthesis of the effectiveness of different feedback parameters was considered inadequate. There were no clear indications as to which specific sensorimotor tasks or target populations might benefit from visual feedback, and where it should be avoided. Therefore, this review reported current trends regarding visual feedback regimes and their effectiveness in the research literature, but it could not provide strong evidence concerning specific feedback parameters. Moreover, when assessing the strength of evidence for or against the specific feedback design used, most included studies had either high concern according to ROB 2 or consisted of relatively small sample sizes per intervention group. As such, the described results should not be taken as definitive evidence, but rather as indications to take into consideration for guiding future research or practical implementation. For these reasons, we cannot give specific recommendations for practical SAFT system design and will instead summarize general considerations based on the designs and recommendations in the literature as well as giving theoretical guidelines to inform future research on SAFT system design.

By employing a strict search procedure specifically narrowed to sensor-based visual feedback, we set out to reduce the breadth of the study scopes *a priori*. These restrictive definitions were intended to facilitate objective evaluation but do not constitute a theoretical consensus. The exclusion of bimanual tasks, for example, was not based on research showing that these movements are necessarily simple tasks, but instead was a result of conservatively avoiding potential interference when including semi-complex tasks. Also, the boundaries between some other reported categories (e.g., concerning experience levels) should only be interpreted as rough indicators. Finally, the restriction to sensor-based feedback excluded functionally identical but non-sensor-based designs. For example, applying body-mounted laser pointers does not utilize sensors but provides the exact same information as a motion sensor and a display [cf. Stien et al. (47)]. On the other hand, raw video replay was included [e.g., Benjaminse et al. (20)] because of the camera sensor, which does not necessarily provide different information than a physical mirror [e.g., Roy et al. (48)].

While we believe we have covered the most important parameters in the design of visual feedback, there may be other important design variations in the remaining body of research beyond our search parameters and the three searched databases, especially in databases more related to sports. Based on the results shown here, we would not expect subsets with sufficient homogeneity to allow generalizable quantification of the benefits of specific feedback parameters even with a larger set of included studies. Including simple movement tasks, which tend to have more standardized testing and outcome measures, would not help with our main research question either because previous research has shown that the effects of feedback do not generalize to complex tasks (13–16). Be that as it may, our sample consisted of various settings in which visual feedback was used effectively, indicating that further usage and study of visual feedback seems warranted: In certain settings, visual feedback can have a positive impact, both on the immediate effects during training and on the learning and retention of complex sensorimotor tasks over longer periods of time.

### Feedback regimes in the literature

We have seen a strong focus on knowledge of performance rather than knowledge of results. This may be explained by the fact that knowledge of results is often readily available (e.g., by looking at the point where a thrown ball has landed), so SAFT systems are not required in these cases. Moreover, designing concurrent knowledge of results may be more difficult and may not even make sense in non-continuous tasks. Indeed, the only case where we have seen concurrent knowledge of results was a cycling task where the result (total distance covered) is continuously updated. The benefits of KR or KP feedback have been discussed extensively in the literature, suggesting that it is a crucial aspect and that it should be considered when comparing one feedback intervention to another (49). However, there may be task goals and feedback regimes where the distinction is not so clear, particularly when execution of a prescribed movement without spatial error is the desired result [e.g., Koritnik et al. (39)].

Regarding the timing of feedback, we have seen little variation in feedback delay, with most feedback being simply described as concurrent or terminal. Sigrist et al. (17) concluded in their review that concurrent feedback is more beneficial as task complexity increases, so this could serve as a guiding principle. Anson et al. (35) argued that visual feedback is better for slow movements because visual processes take longer compared to proprioception. From this perspective, feedback delay is a spectrum rather than a binary property. This seems to be in contrast with the prevailing definition of concurrent or terminal feedback. We also note that in both concurrent and terminal feedback, delays in feedback could theoretically be added to encourage independent self-assessment and error prediction by the learner.

We found that feedback frequency was sometimes not reported, or at least not as a deliberate choice. As mentioned before, a reduced frequency could also be the result of tests during the intervention period. This, of course, should be taken into account when interpreting a feedback intervention from a study or using it in practice, as a different efficacy might be observed if the feedback training is not interspersed with non-feedback tests. In addition, strategies such as self-selected or error-based feedback could lead to an implicit, individualized fading mechanism, that promotes, for example, higher involvement and better transfer (50). If increased competence in the movement task through learning leads to fewer feedback requests or fewer errors exceeding the defined threshold, then this will effectively lead to less feedback received over time, as indicated by the vast discrepancies between average and maximum feedback frequencies in these regimes [e.g., in Post et al. (28)].

Feedback can be presented at different levels of abstraction and reliability. This may include, for example, ambiguities in representation, rounding of scores, combining multiple scores into one score, or over time (i.e., changing the resolution or specificity of the feedback). This can make it more difficult for the subject to interpret the results, introduce a threshold below which errors are imperceptible, or otherwise weaken the link between the measured quantity and the information conveyed to the subject. An example of deceptive feedback was given in Jones et al. (38), which is also a good example of using two different levels of abstraction: In addition to the more precise performance feedback provided by displaying distance traveled as a number, increased speed was also encoded in a complex graphical representation by moving an avatar faster through the environment. Taken in isolation, such complex feedback would not allow accurate differentiation of small changes in speed over time.

Finally, the most versatile parameter for visual feedback is the content of the graphical representation itself. We saw some complex graphics, but many of the included studies had relatively simple representations such as numbers, bars, and plots. The choice of visual feedback display format (such as plots, avatars, videos, etc.) seems to matter little. We would have expected much more variance in this area because it is becoming easier to develop such complex graphics and because commercial products with such graphics are ubiquitous, including exergames or virtual and augmented reality devices. This discrepancy could be explained by visual feedback becoming too complex for the learner to interpret effectively, or by potential confounding factors introduced with complex graphical representations that encode multiple variables simultaneously. Having said that, we have not seen any cases where the authors explicitly stated that the feedback was too hard to understand for the participants. None of the graphical representations were deemed too complex, and none of the quantities too abstract for the participants. As a result, we do not see a reason to restrict these parameters *a priori*. However, we should point out that the number of parameters conveyed at once were always rather small (i.e., at most three). It is not quite clear whether this was a purely scientific decision to control what the participants focus on, or whether this is a feedback design decision because participants may not be able to process or select from too much information at once. We would only expect the latter point to play a big role for concurrent feedback, since in the case of terminal feedback, there is ample time for the participant to study the information and select the most relevant parts in the terminal condition. A possible exception to the generally low number of parameters is present in video feedback: Depending on one's perspective, the scene can be interpreted as one parameter conveying the general silhouette or posture of the whole body, or it can be interpreted as containing a plethora of parameters including limb positions and joint angles. This might also explain the recommendations to guide the participants' focus with appropriate instructions, as this would affect the effective numbers of parameters to interpret.

We should also point out that the main goal of SAFT systems is to be beneficial for overall training, and comprehensibility of the provided feedback is only one aspect of this. It is unclear to what extent the feedback needs to be cognitively processed at all for it to help with the operationalization of certain movement parameters. After all, even if subjects find the visual feedback confusing or do not quite understand it, the feedback could in principle still have a positive effect because some (negative) patterns are still recognizable. This is more apparent in sonification, where understanding the parameterization may be more difficult than hearing when something about the movement is out of the ordinary. Another possible explanation for the relatively low diversity in the graphical content of the feedback are the rather uniform objectives of the feedback regimes we encountered: The feedback regimes were generally focused on direct error correction (with the error in question being directly related to the study outcome measures). Other possible objectives of feedback, such as guided exploration of the task-space through targeted variation of task and feedback parameters, remain largely uncharted. A more in-depth theoretical analysis of the movement tasks and training goals according to the four task-space learning mechanisms could encourage the examination of other feedback objectives.

### Implications for the practical application of SAFT systems and future research

Implications for the application of SAFT systems in practice remain largely speculative. The main challenge to practically apply SAFT systems lies in identifying effective feedback regimes for specific sensorimotor tasks, and specific populations at specific stages of learning. The effectiveness of concurrent feedback may depend on the complexity of the movement as well as the complexity of understanding the task requirements. The optimal modality may depend on gender, speed of movement, and how large a movement is (i.e., visual discernability). There is some evidence that visual feedback is better suited for spatial task aspects (as opposed to temporal tasks), but Sigrist et al. (42) mentioned that this may be an artefact of simplicity of feedback design. In other words, designing intuitive feedback may be more straightforward if it has the same modality as the movement aspect, but that does not mean that otherwise a good design is impossible to find or that this feedback is inherently more effective. There may also be a tradeoff between feedback simplicity and the amount of information conveyed. Video feedback in particular may be too complex for the user, so additional, carefully formulated instruction is required. This guidance should ideally direct the user to correct the error and not just give information about the error, which necessitates a comprehensive understanding of the task and the involved control parameters. Finally, feedback can encourage the user to increase performance, but the effectiveness of this may be highly dependent on the user's preferences or skill level. The feedback should thus ideally be highly individualized and adaptive. When the motivational aspect is the main goal of the feedback, then the feedback regime might be regarded as successful even if it does not affect the overall training efficiency, as long as it does not hinder progress either.

In our opinion, the current research on feedback for complex skill learning does not support any sweeping statement for or against specific feedback regime parameters in practice. In this regard, not much has changed since the call for more intensive research on complex skill learning from Wulf and Shea (14) in 2002. It looks like visual feedback for complex movements at least does not lead to worse learning outcomes in most cases even if no explicit fading was implemented, provided that this is not due to publication bias. This lack of negative outcomes stands in contrast to feedback on simple movements [cf. the guidance hypothesis (11, 12)], which we interpret as corroborating Wulf and Shea's warning against using results from feedback studies with simple movement tasks to inform the feedback design for complex skills.

Whether visual feedback shows a significant positive effect or no significant effect at all seems to depend on the situation—how much this concerns the design of the feedback regime, the movement task, or the characteristics of the participant cannot be said with any certainty based on the current scientific literature. To better explain and predict the effectiveness of feedback in certain settings, standardized evidence is needed, so that a statistical meta-analysis that compares similar settings with low risk of bias becomes feasible. To this end, we call for future research to focus on obtaining clear definitions on what constitutes a complex coordination task and ideally finding task-category-dependent standardized coordination tests that can be utilized as main outcome parameters in different studies. After establishing a solid basis to build upon, systematic experiments varying only single parameters of the provided feedback for specific tasks would have the potential to produce prescriptive feedback design recommendations. Furthermore, generalizability of results from one outcome of interest to others in the context of augmented feedback training should be investigated: For example, it is not clear at the moment whether specific feedback design parameters, such as a reduced feedback frequency, would have the same effect in training for better endurance-running economy and training for increased weight-lifting performance. Interestingly, this need for more uniform, fundamental research on complex movement task learning with feedback mirrors the conclusion reached by Kal et al. (10) in a systematic review comparing the benefits of the implicit and explicit motor learning. This is a clear indication that this problem is not confined to feedback design studies, but rather points to a systematic issue with the design of trials investigating complex movement tasks in general, specifically the lack of trial and reporting guidelines as suggested by Kal et al. While there are useful reporting checklists for exercise studies, such as the Consensus on Exercise Reporting Template (CERT) (51), these checklists are not specific to feedback studies and only cover the reporting rather than the design of studies.

### Theoretical considerations

In the absence of evidence-based guidance, we fall back on the theoretical background to inform future SAFT research to the best possible extent. First and foremost, it should be kept in mind that SAFT systems cannot be designed without considering the characteristics of the task and the instruction regime. Even if no explicit instructions are given to the learner, the way the feedback is presented during or after task execution potentially influences the learner's (implicit or explicit) task goals. As outlined in the introduction, SAFT system designers need to be aware of the subtleties of the well-established and researched motor learning approaches that lie between discovery learning and prescriptive, explicitly instructed learning. Only then can the designer leverage the real potential of systems to systematically assist motor learning during task space formation, exploration, differentiation, and (de-)composition. This is particularly important because instructions and feedback can cause shifts in attentional focus and influence learner motivation, triggering or hindering the learning of task specifics [e.g., compensatory effects (52)]. Unfortunately, the complexity of retrieving the correct instruction and feedback rises with the complexity of the task space. To tackle this issue, a structured approach to task understanding seems necessary. Naturally, domain specific knowledge, e.g., from experts in the field, in addition to evidence from similar previous research could provide a good basis for potentially fruitful feedback regimes. Complementary, functional task analysis (53) seems to be a well-suited approach to guide the identification of structure and functionally relevant features of the sensorimotor task without forcing the user to adopt a specific theoretical stance. Even if naturally the focus, functional assignments for specific modalities of the task's (sub-)actions are not limited to the biomechanical domain but can also be derived from anatomical, physiological, coordinative, perceptual, mental, or tactical perspectives on the sensorimotor task. As Hossner et al. (53) noted, these further functional justifications are based on the fact that a learner's perceptual-motor skills and psychological competencies shape individual task spaces. Hence, functional task analysis seems particularly suitable for the design of SAFT systems, as it automatically distinguishes (functionally irrelevant) style aspects from (functionally relevant) errors in the individual task solution. Both can be incorporated into the design of feedback—the latter as feedback that should be given to ensure correct and functional task solutions, the former as feedback that should be avoided to keep individual freedom and compensation potential high for the motor system and increase its robustness. Once the task space and relevant control variables are identified, the designer can begin to define the intended objectives of the feedback and instructions.

To define the intended objectives of the SAFT, a broad examination and prioritization of the potential benefits of feedback in the target setting is required. We describe some of these potential benefits for visual feedback here, but this list is by no means exhaustive. First, feedback can provide benefits simply by reducing monotony or making the learner more aware of their learning progress, which can, in turn, increase motivation (54). Second, feedback can be used to alter the goal-specifications or shift attentional focus (55). For example, adding an accuracy score in a throwing task might shift the learner's goal: Instead of trying to maximize the power output, the desired result might become movement precision or correct form, guiding the learner closer to an optimal solution. Such feedback may be necessary to guide the learner out of a local optimum in the task-space (4) or to encode variables related to injury risk in the optimization of a movement solution. Third, feedback could focus only on its immediate effect and not on lasting improvements. For example, correct posture and movement execution may be important factors for safety during strength and endurance training. In this case, it may even be beneficial to provide feedback to improve these parameters during each single training session, provided that the exerciser never has to perform these tasks without feedback, and they rather serve as basic building blocks for other skills. Fourth, visual feedback can be easily ignored by looking away, even if this is obviously not considered its primary intent. This may, however, be an advantage of visual feedback over other feedback modalities, as it allows for a form of self-selection that has been reported to increase the effectiveness of feedback and motivation (50). For an even more detailed discussion of the effectiveness of different types of feedback, we refer the reader to the pertinent review by Sigrist et al. (17). Since the intended objective of a feedback is critical for the design of the feedback regime, we additionally refer the reader to Table 1 in Hossner and Zahno (5), where the specific roles of variance in different motor learning mechanisms are summarized.

There is not necessarily a fixed feedback regime that is optimal for all individuals. The optimal feedback strategy might even depend on the individual's daily mood, motivation, or physical condition, and it might change over a single training session with the level of fatigue. In addition, different aspects of the same task may be optimized in different ways, and tradeoffs could occur. For example, injury-prevention, speed, and jump height in volley spikes may be mutually contradicting goals that result in different optimal movement executions depending on the importance placed on each aspect.

Once a promising solution is found, a well-designed intervention study with fair controls is recommended to validate the effectiveness of the feedback intervention. If motivation is a primary objective of the feedback, even a null effect on learning rates may be considered a positive outcome, as it could mean that the motivational benefits can be reaped without impeding training progress. On the other hand, if the feedback-guided intervention is aimed at learning real-world skills in a training setting, transfer tests are needed to validate the effectiveness of the designed intervention, or at least, according to Teran-Yengle et al. (44), some sort of formal documentation of carry-over to normal life. When testing a novel training intervention with feedback, we strongly recommend three intervention groups: One with the novel training intervention with feedback, one with the novel training intervention but without feedback, and one as a classical control (no intervention or reference intervention). With such a design, the study can not only validate the effectiveness of the intervention, but it may also show the extent to which the outcome was influenced by the feedback provided.

### Proposed strategy for SAFT system design in future research

Based on the literature reviewed and the theoretical considerations, we propose the following general strategy for designing SAFT systems in a scientific setting: First, clearly define the intended objectives of the SAFT. Second, conduct a functional task analysis to clearly identify functionally relevant control variables and error mechanisms. Third, determine options for initial feedback solutions based on prior research and domain-specific knowledge. Fourth, if needed to make an evidence-based decision, conduct small pilot studies to choose among different parameter options. Fifth, conduct a well-designed comparative study that includes transfer testing and a single clear main outcome measure. For novel training interventions with feedback, two control groups may be optimal: one with the training intervention without feedback, and one that does not receive the intervention. For established training interventions with novel feedback, a single control group getting the same intervention without feedback is sufficient. In both cases, we do not recommend designating a group receiving different feedback as the control group, unless the utilized feedback can be regarded as the gold standard in that setting. This procedure should support investigation of the potential benefits of a developed feedback intervention in practice as well as answering the question whether the feedback itself made a significant positive contribution to the overall outcome.

### Conclusion

We compiled significant findings, utilized feedback regimes, and recommendations from a set of 26 studies on visual feedback in complex sensorimotor tasks with healthy adults. Although the current evidence base is insufficient to derive clear rules for or against the use of specific feedback regimes in complex sensorimotor tasks, the findings outlined in this review and the referenced research can serve as a basis for the initial steps in the process of developing a feedback regime for learning sports-related skills. Consideration of the properties of the sensorimotor task, the task instructions, the feedback regime, and the intended objectives of the feedback is critical. Because the evidence in the literature does not form a strong basis for an evidence-based feedback design guidance, the proposed strategy for future sensor-based augmented feedback training research is instead based on statements in the literature as well as the theoretical background. These considerations are only meant to inform feedback intervention studies in the interim. Standardized study design and reporting guidelines for motor learning research on complex movements, compiled by experts on motor control, are needed to direct future research in a way that will lead to a stronger scientific foundation that can adequately inform design decisions for sensor-based augmented feedback systems in practice.
